# Repeated isolation of tick-borne encephalitis virus from adult *Dermacentor reticulatus* ticks in an endemic area in Germany

**DOI:** 10.1186/s13071-019-3346-6

**Published:** 2019-03-12

**Authors:** Lidia Chitimia-Dobler, Giulia Lemhöfer, Nina Król, Malena Bestehorn, Gerhard Dobler, Martin Pfeffer

**Affiliations:** 1 Bundeswehr Institute of Microbiology, Neuherbergstrasse 11, 80937 Munich, Germany; 2 Parasitology Unit, University of Hohenheim, Emil-Wolff-Str. 35, 70599 Stuttgart, Germany; 3 Institute of Animal Hygiene and Veterinary Public Health, Faculty of Veterinary Medicine, University of Leipzig, An den Tierkliniken 1, 04103 Leipzig, Germany

**Keywords:** *Dermacentor reticulatus*, TBE virus, Saxony, Germany

## Abstract

**Background:**

Tick-borne encephalitis (TBE) virus is transmitted to humans and animals through tick bites and is thought to circulate in very strictly defined natural environments called natural foci. The most common tick serving as a vector for the TBE virus in central Europe is *Ixodes ricinus*; it is rarely found in other tick species and in *Dermacentor reticulatus* it has, so far, only been reported in Poland.

**Methods:**

Between autumn 2016 and spring 2018 ticks were collected by the flagging method in a new TBE focus in the district of northern Saxony, Germany, outside the known risk areas as defined by the national Robert Koch Institute. Ticks were morphologically identified and tested in pools for the presence of TBE virus using a real-time RT-PCR. TBE virus from positive pools was isolated in A549 cells, and the E gene sequences were determined after conventional RT-PCR, followed by a phylogenetic comparison.

**Results:**

TBE virus was detected in 11 pools, 9 times in flagged adults *D. reticulatus* (*n* = 1534; MIR: 0.59%, CI: 0.29–11.3%) and only twice in *I. ricinus* nymphs (*n* = 349; MIR: 0.57%, CI: 0.02–2.2%). All other ticks, *I. ricinus* males (*n* = 33), females (*n* = 30) and larvae (*n* = 58), as well as 5 *I. inopinatus* (2 females, 3 males) and 14 *Haemaphysalis concinna* (3 females, 11 nymphs), tested negative for TBE virus. TBE virus was not detected in *I. ricinus* during the summer, when *D. reticulatus* is not active. Sequence comparison of the entire E gene of the isolated virus strains resembled each other with only 3 nucleotide differences. The most closely related viral sequences belong to TBE virus strains from Poland and Neustadt an der Waldnaab (county of Neustadt an der Waldnaab, Bavaria), approximately 200 km east and 200 km south-west of the new focus, respectively.

**Conclusions:**

TBE virus was found in northern Saxony, Germany, with similar MIRs in *D. reticulatus* and *I. ricinus*, indicating that *D. reticulatus* plays an equal role to *I. ricinus* in virus circulation when both tick species are sympatric.

## Background

Tick-borne encephalitis (TBE) virus (genus *Flavivirus*, family *Flaviviridae*) is the etiological agent of TBE, the medically most important member of the tick-borne serocomplex [1]. At present, three subtypes of TBE virus are recognized: the European (western) subtype (TBEV-EU), the Siberian subtype (TBEV-Sib) and the Far-Eastern subtype (TBEV-FE) [2]. Russian virologists have claimed two new subtypes, both isolated in the Lake Baikal region in Siberia, which are genetically more distant to each of the three currently accepted TBE virus subtypes [3].

Of the 54 species of ixodid ticks known from the western Palaearctic [4], eight species from three genera are known to be able to transmit TBE virus, and so far the virus has been isolated from at least 14 other species (see references in [5]). *Ixodes ricinus*, the most commonly encountered European tick species, is considered to be the major vector of the European TBE virus [6, 7]. Lichard & Kozuch [8] were able to show TBE virus persistence and transmission to white mice by *Ixodes arboricola*, which is considered to be a secondary amplifying vector of TBE virus in wild rodent populations [9]. *Ixodes persulcatus* is the main vector tick species known to transmit TBEV-Sib and TBEV-FE [10]. *Haemaphysalis concinna* is also a known vector of TBE virus [11, 12]. Nosek et al. [13] experimentally proved the vector competence of *Haemaphysalis inermis* for TBE virus. *Ixodes gibbosus* is a marginal vector in the Mediterranean region [14]. In addition, TBE virus has been found in other tick species but transmission has not been demonstrated, for example in *Ixodes frontalis* [10, 15, 16]. The virus has been isolated in the Czech Republic from females and nymphs of *I. hexagonus* infesting a hedgehog [17], as well as in Croatia from a pool of three females removed from a red fox [18]. *Haemaphysalis punctata* has also been associated with TBE virus [19].

The genus *Dermacentor* (family Ixodidae) includes 35 species and has a worldwide distribution, except for Australia [20]. *Dermacentor* species are found mostly in Europe, Asia and North America [21]. In Europe, two species, *D. reticulatus* (‘the ornate dog tick’) and *D. marginatus* (‘the ornate sheep tick’), as well as one in Asia, *D. nuttalli*, are associated with TBE virus. Both *D. marginatus* and *D. reticulatus* are competent vectors of this virus [22–24]. The role of *Dermacentor* ticks in the circulation of TBE virus in the environment is, however, unclear and poorly studied [25, 26]. *Dermacentor reticulatus* appears to be spreading and its distribution area and its population density have been increasing during recent decades [25–29]. In eastern Poland, the average prevalence of infection with TBE virus found in *D. reticulatus* was 10.8%, which is considerably higher than the prevalence found in *I. ricinus* (1.6%) [30]. The prevalence rate in *D. reticulatus* ticks from Białowieża Primeval Forest was similar (1.58%) [31] to that in *I. ricinus* (1.3%) [32], which is in line with the case from Moldova (*I. ricinus*, 3.8%; *D. reticulatus*, 3.9%; but *Haemaphysalis punctata*, 8.8%) [33]. However, the dynamics of TBE virus in the *D. reticulatus* tick population in TBE natural foci has not been studied so far and no data on TBE virus circulation in *D. reticulatus* in central Europe are available.

Every active tick stage can be infected with TBE virus [34], and approximately 0.1–5% of the ticks in an endemic area carry the virus [6]. As larvae and nymphs of *D. reticulatus* are nidicolous and adults exophilic (non-nidicolous), only the female *D. reticulatus* ticks are considered to play a major role in the virus transmission to larger mammals including humans, while in case of *I. ricinus*, the nymph is the key stage in viral transmission. Tick males, which either do not feed at all or parasitize only for a short time, might also be involved in virus transmission. Investigations of the viral load of either nymphs, female or male ticks from German natural foci by means of quantitative real-time RT-PCR did not show any significant differences in virus load between the respective stages and sexes (Dobler et al., unpublished results). However, in a Russian study the researchers stated that male ticks appear to be responsible mainly for asymptomatic infections because of their lower virus load and short time of sucking, and thus can contribute to host and population immunity [35]. TBE virus invades all tick tissues, including the salivary glands and ovaries [36], thus it may be transmitted in various ways: (i) take up of the virus during viremia of the host; (ii) *via* co-feeding (direct uptake of the virus from one tick feeding on a non-viremic host in close proximity to a tick harboring the virus); (iii) transstadial, (from one life stage to the next life stage after molting); (iv) transovarial (vertical from female tick to her eggs); (v) transsexual (between male and female during mating on the host); and (vi) infection of the next host *via* saliva (reviewed in [5]).

In this study we describe a new natural TBE focus in Germany and the phenology of TBE virus in *D. reticulatus* for a period of three seasons.

## Methods

### Study site

**Fig. 1:**
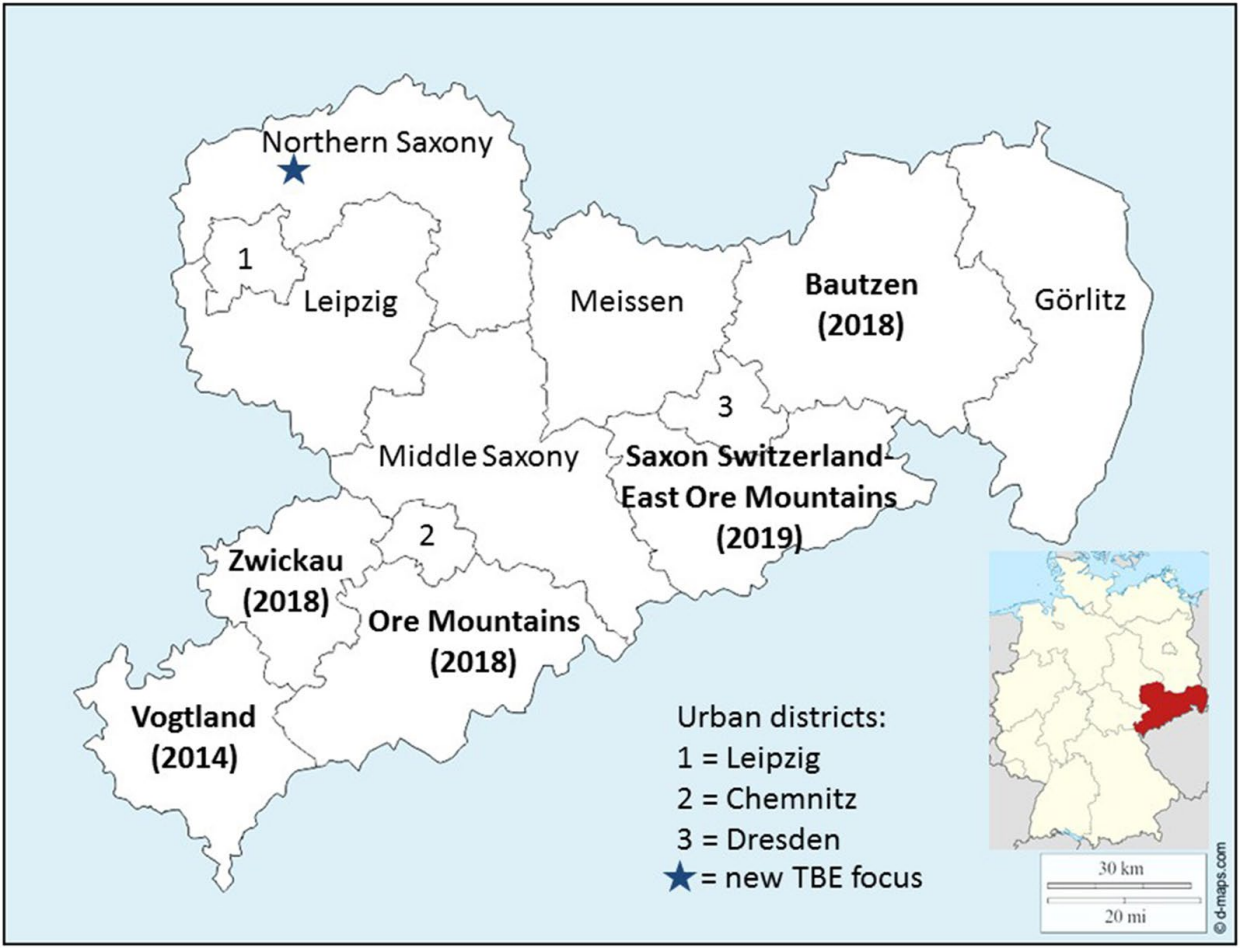
A map of Saxony showing all counties. Names of counties considered as TBE risk area according to the federal Robert Koch Institute are named with bold lettering showing the year since they were considered TBE risk area in parentheses. The new TBE focus described here is indicated by an asterisk. At the time of our study it was about 150 km north of the next known risk area

In July 2016, the first ever human TBE case was reported from the district Northern Saxony in the German Federal State of Saxony, so far classified as a TBE non-endemic area (Fig. 1). The exact location where the infection most likely took place could be identified with the patient’s information. The landscape is dominated by a rather young mixed forest consisting of birch, oak, maple and pine trees, the latter up to 80 years-old, while the deciduous trees are mostly younger than 20 years of age. In the undergrowth, blueberries, blackberries and raspberries can be found. The forest is interrupted by patches of meadow and surrounded by larger stretches of agricultural land. The nearest human settlement is a small village (Battaune), which is about 500 m away; no industrial areas or waste disposal sites are located within this area. Only a small area surrounding a demolished house is used as illegal dump site for household trash. There are 11 rather large areas of livestock holdings within a radius of approximately 1 km of the flagging area.

### Tick collection and identification

All specimens were collected as questing ticks by flagging as part of a tick-borne encephalitis (TBE) programme in Battaune, Federal State of Saxony, Germany, at the end of September 2016, in February, April, July and September 2017, and April 2018. The ticks were kept alive in 50 ml plastic tubes until identification and further testing in the laboratory. Ticks were identified to species level using the morphological characters according to Feider [37], Filippova [38] and Estrada-Peña et al. [39].

### Nucleic acid extraction and PCR

Ticks were processed in pools of 10 nymphs or 5 adults (females or males) per pool. Ticks were homogenized using 1 ml minimum essential medium (MEM, Invitrogen, Karlsruhe, Germany) containing an antibiotic-antimycotic solution (ABAM, Invitrogen) using a Fast Prep Savant FP120 tissue lyser (Bio101, Vista, USA), with three rounds at speed 6.5 for 30 s each. Total nucleic acid was extracted using the MagNA Pure LC RNA/DNA Kit (Roche, Mannheim, Germany) in a MagNA Pure LC instrument (Roche, Mannheim, Germany) according to the manufacturer’s instructions using 200 µl of the tick homogenate. The total nucleic acid was extracted in 50 µl and a 5 µl aliquot was tested for TBE virus RNA using real-time RT-PCR (RT-qPCR; [40]).

### Virus isolation

A 100 μl-aliquot of the supernatants of each crushed RT-qPCR-positive tick pool was added to an 80% confluent cell culture of A549 cells (human lung carcinoma cells, German Collection of Microorganisms and Cell Cultures, DSMZ, Braunschweig). The supernatants were kept at -80 °C until they were used undiluted and in a dilution of 1:5 and 1:10 for virus isolation. After 1 h of incubation at 37 °C, the supernatant was pipetted off and the cells were washed 5 times with MEM containing ABAM. Five milliliters of MEM containing 5-fold concentrated ABAM and 3% fetal calf serum were added. Cells were incubated for up to 7 days at 37 °C and observed daily for the occurrence of cytopathogenic effect (cpe). In case of more than 50% cpe, the supernatant was taken and tested by RT-qPCR for TBEV as described. In case of no cpe, culture supernatant was taken after 7 days of incubation and also tested for growth of TBEV by RT-qPCR. No subcultures were conducted. From the isolated TBEV strains E genes were sequenced for confirmation (see below).

### Sequence analyses of TBE virus E-gene

The E-gene was amplified with a conventional PCR directly from positive tick nucleic acid extractions [41]. This RT-PCR amplified a 1687 bp fragment which was then sequenced using the outer primers and an additional internal sequencing primer as described previously [41]. The products were purified after gel electrophoresis and processed as described [42]. All sequence data were processed using the program Geneious v.9.1.5. A *de novo* assembly was performed using the three chromatograms obtained from GATC (Eurofins Genomics, Ebersberg, Germany) for each positive sample. Nucleotides with an estimated error higher than 1% were trimmed. Subsequently the sequences were cut to 1488 bp, the exact length of the envelope gene sequence. A ClustalW alignment with several other E genes from selected isolates was performed and a phylogenetic tree was generated using the PhyML algorithm [43].

### Statistical analyses

The minimum infection rate (MIR) was calculated for pools of 10 nymphs or 5 adults. It was assumed that only one tick specimen in the pool was infected if the pool tested positive. Fisher’s test was used to compare the prevalence levels for significant independence using GraphPad Prism v.4 (Graph Pad Software, San Diego, CA, USA). The significance threshold was set at *P* = 0.05. Confidence intervals (95% CI) for the prevalence of pathogens were determined by the modified Wald method.

## Results

Overall, 2023 ticks belonging to four species (*Dermacentor reticulatus*, *Ixodes ricinus*, *I. inopinatus* and *Haemaphysalis concinna*) were collected by flagging (Table 1). In September 2016, 996 ticks were collected during 3 consecutive days: 816 *D. reticulatus* (502 females and 314 males); 173 *I. ricinus* (12 females, 12 males, 91 nymphs and 58 larvae); 4 *I. inopinatus* (2 females and 2 males) and 3 *H. concinna* nymphs. Of the 174 pools, TBE viral RNA was detected in 3 pools of *D. reticulatus* (1 female and 2 male pools) and 1 *I. ricinus* nymph pool.

**Table 1:** Tick species and life stages collected during the study and number of TBE virus positive pools

Collection time	*Dermacentor reticulatus*			*Ixodes ricinus*					*Ixodes inopinatus*			*Haemaphysalis concinna*			Total *N* (*n*)
	Female *N* (*n*)	Male *N* (*n*)	Total *N* (*n*)	Female *N* (*n*)	Male *N* (*n*)	Nymph *N* (*n*)	Larva *N* (*n*)	Total *N* (*n*)	Female *N* (*n*)	Male *N* (*n*)	Total *N* (*n*)	Female *N* (*n*)	Nymph *N* (*n*)	Total *N* (*n*)	
IX 2016	502 (1)	314 (2)	816 (3)	12	12	91 (1)	58	173 (1)	2	2	4	0	3	3	996 (4)
II 2017	234 (3)	169	403 (3)	3	2	7	0	12	0	1	1	0	0	0	416 (3)
IV 2017	31 (1)	28	59 (1)	1	4	22	0	27	0	0	0	0	0	0	86 (1)
VII 2017	0	0	0	6	10	150	0	166	0	0	0	3	7	10	176
IX 2017	89 (1)	69	158 (1)	0	2	24 (1)	0	26 (1)	0	0	0	0	1	1	185 (2)
IV 2018	57	41 (1)	98 (1)	8	3	55	0	66	0	0	0	0	0	0	164 (1)
Total	913 (6)	621 (3)	1534 (9)	30	33	349 (2)	58	470 (2)	2	3	5	3	11	14	2023 (11)

*Abbreviations*: N, number of collected ticks; n, number of TBEV-positive pools

In 2017, 863 ticks were collected during the year, from which 620 *D. reticulatus* (266 males and 354 females), 231 *I. ricinus* (18 males, 10 females and 203 nymphs), one *I. inopinatus* (male), and 11 *H. concinna* (3 females and 8 nymphs) were identified. Details regarding tick species activity are presented in Table 1. From specimens collected in February, 90 pools were tested and 3 *D. reticulatus* female pools were positive. From ticks sampled in April, 16 pools were tested: 12 *D. reticulatus* pools and 4 *I. ricinus* pools, with 1 *D. reticulatus* female positive pool. From ticks from July, 27 pools were analysed: 22 *I. ricinus* pools and 2 *H. concinna* nymph pools and 3 individual females, none of which were positive. From individuals collected in September, 38 pools were investigated and 2 pools were positive, 1 *D. reticulatus* female pool and 1 *I. ricinus* nymph pool.

In April 2018, 164 ticks were collected: 98 *D. reticulatus* (41 males and 57 females) and 66 *I. ricinus* (3 males, 8 females and 55 nymphs). Of 33 pools, one male *D. reticulatus* pool was positive.

In total, the detection of viral RNA in 11 tick pools was confirmed by the isolation of virus strains from 3 male and 6 female *D. reticulatus* pools and two pools of *I. ricinus* nymphs.

In general, the minimum infection rate (MIR) for *D. reticulatus* was 0.59% (CI: 0.29–11.3%) and 0.42% (CI: 0.01–1.64%) for *I. ricinus* ticks and 0.57% (CI: 0.02–2.2%) for *I. ricinus* nymphs. There were no statistical differences in the TBE prevalence between *D. reticulatus* ticks and *I. ricinus* nymphs (*P* = 1.000) or ticks (*P* = 1.000). The tested *I. ricinus* ticks were all negative in July 2017 when *D. reticulatus* ticks were entirely absent.

**Fig. 2:**
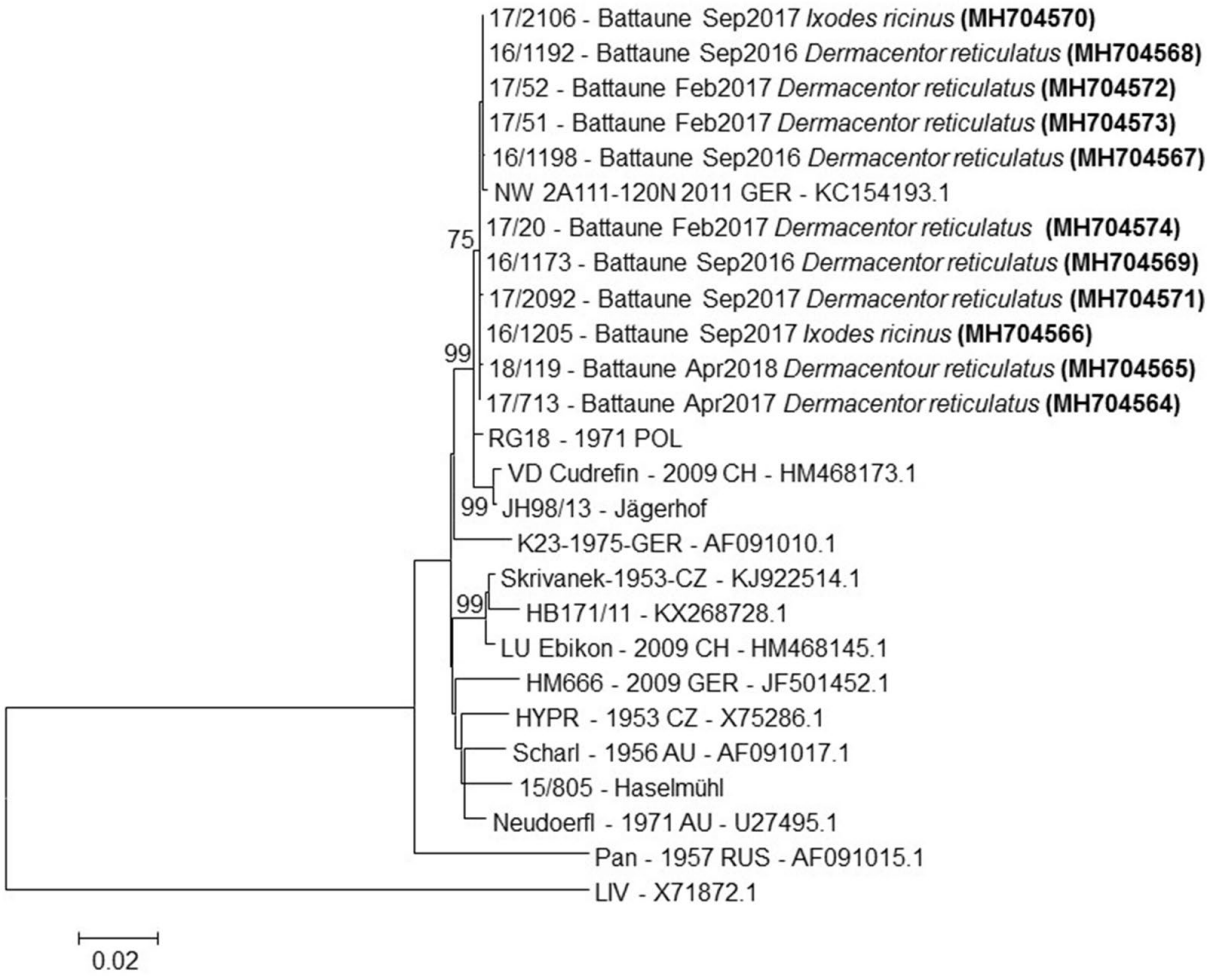
Phylogenetic analysis of complete E gene sequences of European TBE virus strains using louping ill virus (LIV) as outgroup. Nucleotide sequences generated for this manuscript are given in bold and the name of the respective tick species is provided. The countries of origin are given as: GER, Germany; CZ, Czech Republic; AU, Austria; CH, Switzerland; POL, Poland; RUS, Russia. GenBank accession numbers are also provided

A total of 11 E-genes sequences from the positive tick pools were generated and analysed. The phylogenetic tree is shown in Fig. 2. The analysis showed that all virus strains belong to one single genetic cluster of TBE virus. Phylogenetic analysis of the E gene sequences with other available E genes from TBE virus strains of different geographical areas in Europe exhibited a close relationship to TBE E-gene sequences from Poland, southern Germany and Switzerland. The virus strains from *D. reticulatus* ticks showed no difference to virus strains from *I. ricinus* ticks from the focus and from other foci (strain NW-2A-111 from *I. ricinus* from Germany; RG18 from *I. ricinus* from Poland, 1971). The actual sequences of the E genes were also closely related to sequences from Germany (2011) and from Poland (1971).

## Discussion

TBE virus was detected and isolated repeatedly from *D. reticulatus* during three sampling seasons in an area of sympatric occurrence together with *I. ricinus* and, to a lesser extent, *I. inopinatus* and *H. concinna*. Although TBE viral RNA has previously been detected in *D. reticulatus* ticks [30–32] and its capability of transmitting TBE virus has been demonstrated [13], this was a surprising finding. The maintenance of TBE virus by *D. reticulatus* in natural foci in the absence of *Ixodes* ticks was considered highly unlikely [44]; however, *D. reticulatus* was considered to support TBE virus circulation in the presence of *I. ricinus* populations. Our results suggest the opposite, i.e. that *D. reticulatus* is at least of equal importance in the maintenance of TBE virus in this natural focus as (i) we found it throughout our sampling period and (ii) the virus was absent in the *I. ricinus* population during the summer when adult *D. reticulatus* cannot be flagged (see below).

In European parts of Russia where *D. reticulatus* sympatrically occurs with *I. persulcatus*, a genetically closely related species to *I. ricinus*, the virus was also found in both tick species [45]. In contrast to the situation described here, the authors found a sympatric occurrence of *D. reticulatus* and *I. persulcatus* throughout the year while in Germany, as in central Europe, *D. reticulatus* adults do not quest and thus cannot be collected by flagging in summer [28, 46, 47]. Recent reports from Poland support our findings, where TBE virus was detected in 10.8% of 148 *D. reticulatus* and in only 1.6% of 875 *I. ricinus* [30]. As immature stages of all these tick species can feed on the same host at the same time, infection may occur on viremic rodents or during co-feeding on the same small mammal as demonstrated in Udmurtia [45]. As transstadial transmission was also previously demonstrated for *D. reticulatus* [36], the most likely source of the TBE virus infection of these nidicolous immatures are rodents, mainly mice of the genus *Apodemus* and the bank vole (*Myodes glareolus*), known to serve as reservoir hosts for TBE virus [34, 36]. Future attempts must investigate the role of small mammals in the TBE virus transmission cycle at this natural focus. However, the detection and isolation of TBE virus in *Dermacentor* tick species is not very common and the reasons for this are neither known nor understood. The repeated findings of TBE virus positive *D. reticulatus* ticks in three consecutive years, 2016–2018, are notable. It may be speculated that because TBE virus has been recently introduced into this area (as no human TBE case has been reported in this region since 2001 when TBE became a notifiable disease in Germany), it seems very likely that the most abundant tick species became the main carrier of the virus.

It is interesting to note that positive *I. ricinus* nymphs were found only in September, when both *I. ricinus* and *D. reticulatus* activity was high. To explain if this was an accidental occurrence, more studies should be conducted. However, the positive testing of TBE virus might possibly support the theory that the tick species *D. reticulatus* plays a major part in the conservation of this TBE natural focus. This would be consistent with the findings of the Polish research team of Biernat et al. [31] in Poland. This is also mirrored by the equal MIR of *D. reticulatus* and *I. ricinus* nymphs with 0.59% and 0.57%, respectively. Interestingly the total (nymph and adults) *I. ricinus* MIR of 0.42% was very similar to 0.47% found in a recent study investigating the prevalence of TBE virus in the upper Rhine region in 2016–2017, more than 500 km away from the TBE focus investigated here [48].

In contrast to this study, where only two E gene sequences were identical [48], our phylogenetic analyses did not reveal a striking difference between nucleotide sequences derived from either *D. reticulatus* or *I. ricinus* ticks. The branching of our sequences together with sequences from Poland (approximately 200 km east) and Neustadt an der Waldnaab (approximately 200 km south-west) are in line with a general east to west evolution of TBE viruses [49], but they do not provide hints why *D. reticulatus* seems to be the key vector in TBE virus maintenance in this natural focus. The phylogenetic analyses of the E genes show a marked high homology to virus strains from Poland isolated in 1971 and from Germany isolated in 2011. It is surprising that the virus did not show mutations during this long time period. It might be an indication of a rather recent introduction from Poland or South Germany. The data also show that there is so far no adaptation of the TBE strain to different tick species belonging to different tick genera. *Dermacentor* and *Ixodes* separated more than 200 million years ago and therefore are genetically very distant. One would expect that an RNA virus would adapt to such different vectors and reservoirs. The TBE virus detections in *D. reticulatus* in Poland showed a sequence in the C gene, which the Polish group interpreted as specific for the tick species *D. reticulatus*. We did not investigate the C gene as the E gene is thought to be more informative with regards to virus strain separation [49]. The lack of differences in the E genes of the virus strains from different tick species might, however, also be indicative of a recent introduction of this strain into the population of *D. reticulatus*. It will be interesting to follow the genetic evolution of this virus strain during the next years to show whether changes will be introduced in the genome of the virus specific for *D. reticulatus* ticks.

## Conclusions

TBE virus was detected in *Dermacentor reticulatus* ticks throughout their activity season and only occasionally in *Ixodes ricinus* sharing the same area in sympatry. In the warm summer months when adult *D. reticulatus* are not questing and thus cannot be flagged, the virus was not found in active *I. ricinus* of any life stage, further supporting the conclusion that *D. reticulatus* may play an important role in virus circulation in this newly established TBE focus.

## Abbreviations

ABAM: antibiotic-antimycotic
AU: Austria
C: capsid
cpe: cytopathogenic effect
CH: Switzerland
CZ: Czech Republic
E: envelope
GER: Germany
LIV: louping ill virus
MEM: minimum essential medium
MIR: minimum infection rate
POL: Poland
RNA: ribonucleic acid
RUS: Russia
RT-PCR: reverse transcription-polymerase chain reaction
RT-qPCR: reverse transcriptase-quantitative polymerase chain reaction
TBE: tick-borne encephalitis
TBEV: tick-borne encephalitis virus
TBEV-EU: European (western) subtype of tick-borne encephalitis virus
TBEV-Sib: Siberian subtype of tick-borne encephalitis virus
TBEV-FE: Far-Eastern subtype of tick-borne encephalitis virus
